# Rescue karyotyping: a case series of array-based comparative genomic hybridization evaluation of archival conceptual tissue

**DOI:** 10.1186/1477-7827-12-19

**Published:** 2014-03-03

**Authors:** Rashmi Kudesia, Marilyn Li, Janice Smith, Ankita Patel, Zev Williams

**Affiliations:** 1 Program for Early and Recurrent Pregnancy Loss (PEARL), Department of Obstetrics, Gynecology & Women’s Health, Albert Einstein College of Medicine, 1301 Morris Park Avenue, Price Building, Room 474, Bronx, NY 10461, USA; 2 Department of Molecular and Human Genetics, Baylor College of Medicine, One Baylor Plaza, Mail Stop NAB 2015, Houston, TX 77030, USA

**Keywords:** Recurrent pregnancy loss, Miscarriage, aCGH

## Abstract

**Background:**

Determination of fetal aneuploidy is central to evaluation of recurrent pregnancy loss (RPL). However, obtaining this information at the time of a miscarriage is not always possible or may not have been ordered. Here we report on “rescue karyotyping”, wherein DNA extracted from archived paraffin-embedded pregnancy loss tissue from a prior dilation and curettage (D&C) is evaluated by array-based comparative genomic hybridization (aCGH).

**Methods:**

A retrospective case series was conducted at an academic medical center. Patients included had unexplained RPL and a prior pregnancy loss for which karyotype information would be clinically informative but was unavailable. After extracting DNA from slides of archived tissue, aCGH with a reduced stringency approach was performed, allowing for analysis of partially degraded DNA. Statistics were computed using STATA v12.1 (College Station, TX).

**Results:**

Rescue karyotyping was attempted on 20 specimens from 17 women. DNA was successfully extracted in 16 samples (80.0%), enabling analysis at either high or low resolution. The longest interval from tissue collection to DNA extraction was 4.2 years. There was no significant difference in specimen sufficiency for analysis in the collection-to-extraction interval (p = 0.14) or gestational age at pregnancy loss (p = 0.32). Eight specimens showed copy number variants: 3 trisomies, 2 partial chromosomal deletions, 1 mosaic abnormality and 2 unclassified variants.

**Conclusions:**

Rescue karyotyping using aCGH on DNA extracted from paraffin-embedded tissue provides the opportunity to obtain critical fetal cytogenetic information from a prior loss, even if it occurred years earlier. Given the ubiquitous archiving of paraffin embedded tissue obtained during a D&C and the ease of obtaining results despite long loss-to-testing intervals or early gestational age at time of fetal demise, this may provide a useful technique in the evaluation of couples with recurrent pregnancy loss.

## Background

Recurrent pregnancy loss (RPL) is a common though poorly understood condition. Defined as two or more failed clinical pregnancies, RPL affects up to 5% of couples attempting to conceive [1,2]. In addition to the physical burdens, RPL is often an emotionally and psychologically difficult diagnosis for these women and their partners. Diagnosing the cause of a pregnancy loss is important both to determine whether further interventions are indicated, as well as to provide a sense of closure to the patient and her partner.

Chromosomal abnormalities account for up to 75% of first trimester pregnancy losses [3-10]. Given this high frequency, a critical initial step in the evaluation of recurrent pregnancy loss is to perform cytogenetic analysis of the products of conception (POCs) in order to determine whether aneuploidy was the cause of the loss. If the pregnancy loss was due to aneuploidy, then the likelihood of recurrence returns to the age-adjusted baseline, which increases with advancing maternal age [11]. However, if aneuploidy was not the cause, a further work-up is indicated.

Traditionally, cytogenetic evaluation following a pregnancy loss is performed via Giemsa staining of metaphase spreads. This requires culturing cells obtained from freshly collected chorionic villi, and so POCs must be promptly placed in appropriate culture media before cell death occurs. These requirements create many situations in which conventional karyotyping may not be feasible. Examples include when the pregnancy loss occurred a significant time before the POCs could be placed in appropriate culture media (e.g. patients with a lengthened interval between the demise and the time of tissue collection or when tissue is passed at home or at a location where culturing could not be started), when there may not be the necessary culture media or situational awareness required to send the material for karyotyping (e.g. in the case of emergent dilation and curettage (D&C)) or when samples are lost in transit, become infected, or fail to grow during culture.

Due to the high likelihood of aneuploidy in cases of sporadic pregnancy loss, current recommendations by the American College of Obstetrics and Gynecology as well as the American Society for Reproductive Medicine are to obtain karyotype results only after the second or third loss [12]. Recent cost-analyses have supported this recommendation [13,14]. Thus, the POCs from a first or second loss are often not sent for karyotyping, even following a D&C. However, if the patient goes on to have subsequent losses, particularly at earlier gestations precluding successful genomic analysis, the karyotypes from the earlier miscarriage would be informative. Therefore, there is a need to establish a standardized method for retrospectively retrieving cytogenetic information from previous pregnancy losses in a robust manner that could be readily employed in a clinical laboratory.

In the context of freshly collected POCs, array-based comparative genomic hybridization (aCGH) may be equally or even more informative than conventional karyotyping [15-23]. When used in prenatal diagnosis and the evaluation of children with developmental delay, aCGH allows for the detection of copy number variants (CNVs) such as deletions and insertions with a 10-fold increased resolution compared with traditional karyotyping [24-27].

Unlike prior approaches that required PCR amplification of extracted genomic DNA [28], an automated approach has successfully been utilized in tumor genotyping and cytogenetic analysis in oncology. In these cases, aCGH analysis of DNA extracted from paraffin-embedded tumor samples is frequently used, demonstrating the distinct advantage that this technology does not require live cells [29]. Here we sought to extend this approach to analysis of paraffin embedded POCs.

For the purpose of retrospectively or “rescue” karyotyping, we adapted aCGH analysis of DNA extracted from paraffin embedded tissue, a technique widely used in analysis of tumor samples, to analysis of paraffin embedded POCs. This technique provides the potential to obtain detailed cytogenetic information from previously collected paraffin-embedded conceptual tissue. Based on our findings, we propose this technique as a method of obtaining useful cytogenetic information for patients who require karyotype results but have had either no attempts or failed attempts at conventional karyotyping at the time of prior losses.

## Methods

### Patient selection

This was a retrospective case series carried out at an academic medical center. Patients received care under the auspice of the Program for Early and Recurrent Pregnancy Loss (PEARL) at the Albert Einstein College of Medicine, Bronx NY, and were included if they had RPL and had an indication for karyotyping of a prior loss for which traditional cytogenetic evaluation was either not attempted or was unsuccessful. All patients who met these criteria during the study period were included. In each case, the cost of testing was covered by the patient’s insurance. At the time when the pregnancy loss was diagnosed, gestational age was estimated using fetal biometry. Patients underwent D&C or MVA at the discretion of the provider. The research was reviewed and exempted by the Institutional Review Board (2013-2212).

### Tissue collection

In each case, DNA was extracted from twenty slides of 5 μM thickness obtained from the original paraffin embedded blocks produced for routine pathology examination to confirm POC samples at the time of the original D&C. If DNA was of sufficient quality and quantity, aCGH analysis was performed on high-resolution arrays. This approach enables the detection of CNVs as small as 1 Kb (average 7.5 Kb). However, for purposes of detecting simple aneuploidy (the most likely genetic cause for pregnancy loss), such high resolution is not necessary and so if DNA was of poorer quality or quantity (<500 ng measured by Qubit® fluorometric analysis, Life Technologies) a lower resolution analysis was performed. This low resolution analysis could detect CNVs of 17 Kb, 100-fold smaller than the range that would be needed to diagnose cases of aneuploidy.

### DNA extraction

A slide from the formalin-fixed paraffin-embedded (FFPE) block was reviewed by a pathologist and the area of fetal tissue circled. Fetal or chorionic villus cells were macro-dissected from this circled area to reduce maternal cell contamination (MCC) and subjected to DNA extraction using the QIAamp FFPE DNA extraction kit (Qiagen, Valencia, CA). The gDNA concentration was measured using Nanodrop 1000 (Fisher, Waltham, Massachusetts) and requantified by Qubit 2.0 Fluorometer (Life technologies, Carlsbad, CA).

### aCGH

Cases were run on 2 × 400 K CGH arrays (Agilent Technologies) for high resolution testing. Where DNA was insufficient for high resolution testing, cases with less than 300 ng DNA were run on 4 × 180 K CGH array (Agilent Technologies) for low-resolution testing. Five hundred ng genomic DNA from patients and the reference were digested with *Alu*I and *Rsa*I (Invitrogen) for 2 hours at 37°C and labeled using Agilent enzymatic labeling kit according to manufacturer’s instructions. Patient and reference DNA were labeled with Cy5 and Cy3, respectively. Unincorporated nucleotides were removed using Amicon Ultra-0.5 30 K filter column (Millipore, Billerica, MA). Patient DNA and reference DNA were co-hybridized to arrays for 40 hours at 65°C in a rotator (Agilent Technologies) at 20 rpm. The arrays were then washed with Agilent wash buffer and scanned using an Agilent Microarray Scanner. Scanned data from aCGH was extracted using *Feature Extraction* (version 10.10; Agilent Technologies). Extracted data was analyzed using Agilent *Genomic Workbench* (version 7.0; Agilent Technologies) and Agilent *CytoGenomics* (version 2.0; Agilent Technologies). Genomic copy number changes were identified using Aberration Detection Method 1 (ADM-1), an algorithm implemented in *Genomic Workbench* (Agilent Technologies) and visual evaluation. If MCC was suspected based on results, it was reported as such. In cases where testing was performed for MCC, a rate of approximately 5% was noted, which would be unlikely to impact array results. Testing is validated in-house with known positives and negatives. These methods have been previously reported [21,30].

### Statistical analysis

Statistical evaluation was performed using STATA v12.1 (College Station, TX). It was determined *a priori* that to detect a 60% success rate in rescue karyotyping in our sample compared to the reported values of 85% or greater in the largest studies [24,25], with α = 0.05 and 80% power, at least 20 specimens were needed. Descriptive statistics were computed, and are presented as mean ± SD for normally-distributed and median [inter-quartile range (IQR)] for non-normally-distributed continuous data; categorical data are presented as n (%). Bivariate analysis was performed using Mann–Whitney U and Kruskal Wallis tests for continuous data, and the Pearson’s chi-squared and Fisher’s exact tests for categorical data.

## Results

### Demographics

Seventeen women, providing 20 samples, met criteria over a 12-month period (Table 1). The median maternal age was 33 [30-36], while the median paternal age was 35 [30-36]. The median gravity was 7 [4-9], and the median parity was 2 [0-3]. Of these specimens, the median gestational age at the time of the pregnancy loss was 8 weeks [7-10].

**Table 1:** Baseline demographic and clinical background information of patients and specimens

**Specimen ID**	**Pt ID**	**Maternal/paternal age**	**Gravity/parity**	**GA at loss (weeks)**	**Demise to tissue collection (days)**	**Procedure**	**Tissue collection to DNA extraction (days)**
1	1	30/31	G2P0020	6	0	SP	15
2	2	26/27	G7P2052	16	7	D&C	206
3	2	26/27	G7P2052	20	NA	D&C	728
4	3	33/33	G5P0050	8	0	D&C	545
5	4	26/24	G4P1030	7	NA	D&C	1562
6	5	34/36	G14P3-0-11-3	8	NA	D&C	135
7	5	34/36	G14P3-0-11-3	18	NA	D&C	707
8	6	36/36	G8P3053	11	2	MVA	83
9	7	31/35	G9P4054	8	NA	D&C	224
10	8	22/22	G2P0020	8	1	D&C	100
11	9	35/36	G10P3073	8	2	MVA	229
12	10	40/38	G3P0030	9	0	MVA	202
13	11	28/29	G9P2072	17	2	D&C	114
14	12	30/30	G9P3063	10	NA	D&C	313
15	13	45/43	G10P1091	7	14	D&C	63
16	14	37/39	G7P3043	8	NA	D&C	91
17	15	46/46	G13P6258	7	NA	D&C	39
18	16	30/33	G3P0030	8	NA	D&C	3285
19	16	30/33	G3P0030	6	NA	D&C	254
20	17	36/35	G4P0040	10	NA	D&C	264

*Abbreviations:NA* not available, *SP* spontaneous passage, *D&C* dilation and curettage, *MVA* manual vacuum aspiration.

Among the 17 women, 13 (76.5%) had multiple losses without any prior karyotype performed, while 4 (23.5%) had had cytogenetic evaluation of at least a single prior loss. Three patients in the latter category had prior traditional karyotyping attempts that had been unsuccessful, due to failed growth in culture or being lost in transport.

Of the 20 specimens, information regarding the interval between diagnosis of demise and tissue collection was available for 9 (45.0%) specimens; of these, the median interval was 2 days [0-2]. One specimen (5.0%) was passed spontaneously, while 16 (80.0%) were from a D&C and 3 (15.0%) from manual vacuum aspiration (MVA).

### aCGH testing outcome

Eleven specimens (55.0%) were run at full resolution without difficulty (Table 2). If sufficient DNA for traditional analysis was not available, lower-resolution testing was performed. Five (25.0%) samples were run at low resolution; and 4 samples (20.0%) were insufficient for evaluation. In all, results were generated for the majority of cases, 16 of 20 (80.0%).

**Table 2:** aCGH results

**ID**	**aCGH testing interval (days)**	**DNA input quantity (ng)**	**DNA260/280**	**DNA conc (Qubit) ng/ul**	**DLRS**	**Sufficiency**	**aCGH results**	**aCGH interpretation**
1	13	1000	1.97	161	0.3	HR	arr (1-22,X) *x*2	Euploid female
2	12	1000	1.92	85	0.42	HR	arr (1-22,X) *x*2, gain 70Kb at 1q42.13, loss 230Kb at 3q27.2	Female with unclassified CNV
3	15	500	2.02	28.6	0.47	LR	arr (1-22,X) *x*2	Euploid female
4	13	250	2.03	10.4	0.45	LR	arr (1-22,X) *x*2	Euploid female
5	15	1000	1.99	83	0.67	HR	arr (1-22,X)*x*2, gain 660Kb at 5q13.1, gain 330Kb at 5q32	Female with unclassified CNV
6	13	550	1.91	23	0.33	HR	arr mos 7p11.2-q11.23 (55,824,493-75,930,688) x1	Male with mosaic deletion including Williams syndrome chromosome region
7	35	800	2.01	47.8	0.28	HR	arr (1-22) *x*2, (XY) x1	Euploid male
8	14	1000	21.93	446	0.28	HR	arr (1-22,X) *x*2	Euploid female
9	2					I	-	Insufficient fetal tissue
10	2					I	-	Insufficient fetal tissue
11	9	220	1.98	26.6	0.71	LR	arr (1-12,14-22) *x*2, mos (13) x3, mos (X) *x*2, mos (Y) x1	MCC in T13 male v. vanished T13 female twin v. chimeric pregnancy v. true mosaic
12	9	460	1.62	14.9	0.7	LR	arr (1-20,22,X) *x*2 (21) x3	Female with Trisomy 21
13	47	1000	1.9	154	0.26	HR	arr (1-22,X) *x*2	Euploid female
14	27	600	2.01	41.8	0.4	LR	arr (1-22,X) *x*2	Euploid female
15	40	1000	1.91	92	0.34	HR	arr18p11.32q23 (1-78847598)x3;	Female with Trisomy 18
16	37	1000	2.05	94.2	0.32	HR	arr (1-22, X) *x*2	Euploid female
17	41	300	1.9	15.7	0.31	HR	arr 11p11.32q23 (1-135006515)x3	Male with Trisomy 11
18	30		1.99	56.4		I	-	Insufficient fetal tissue
19	1					I	-	Insufficient fetal tissue
20	50	750	1.88	48.4	0.44	HR	arr4p16.3p15.2 (230836-27342422) x1, arr5q33.2q35.3 (154280916-180674029) x3	Male with unbalanced 4p-5q translocation; known paternal paracentric inversion

*Abbreviations:DLRS* Distribution of Log2 Ratio Spread, *CNV* copy number variant, *HR* high-resolution, *LR* low-resolution, *I* insufficient, *MCC* maternal cell contamination, *T13* Trisomy 13.

The longest duration between documented loss of fetal cardiac activity and collection of tissue that resulted in a successful karyotype analysis was 14 days, though this information was not documented for many patients and is often inaccurate due to unknown time of fetal demise. The longest duration between collection of tissue and DNA extraction that resulted in a successful karyotype analysis was 4.2 years (1562 days).

**Figure 1:**
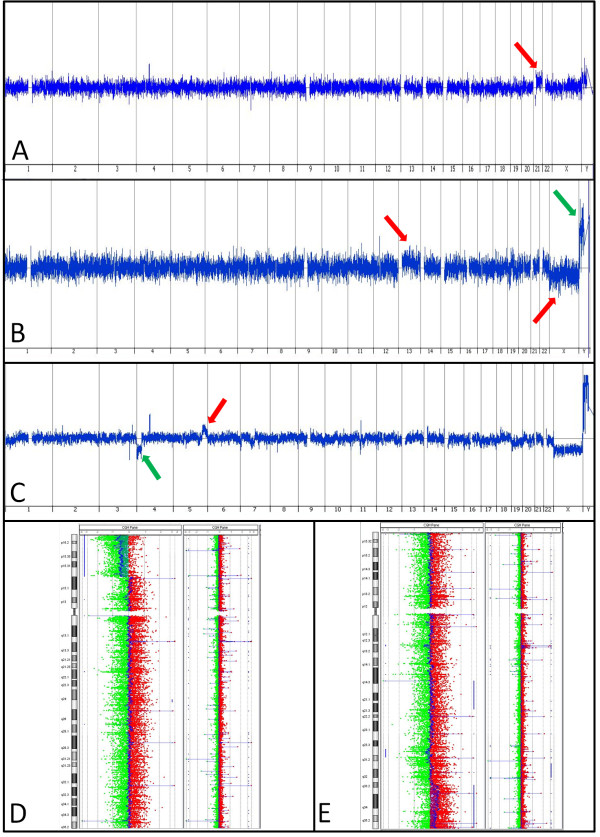
**Selected Array CGH Results. (A)** Specimen 12. Red arrow shows gain of a copy of chromosome 21. **(B)** Specimen 11. Red arrows show mosaic gains of chromosome 13 and chromosome X, green arrow shows a mosaic loss of chromosome Y (a female reference DNA was used in this case). **(C-E)** Specimen 20. C shows the whole genome view of this unbalanced translocation, with the green arrow showing a partial loss of 4p and the red arrow showing a partial gain of 5q. D shows a whole chromosome view of chromosome 4 with a normal case adjacent for comparison to highlight the partial loss of 4p, seen where the DNA signal (in blue) deviates leftward. E shows a chromosome view of chromosome 5, also with a normal case adjacent for comparison, to highlight the partial gain in 5q where the DNA signal (in blue) deviates rightward.

Of the 16 specimens that had sufficient DNA for aCGH analysis, 8 (50.0%) were euploid, and 8 (50.0%) showed copy number variants potentially responsible for the pregnancy loss (Table 2, Figure 1). While an instance of trisomy likely explains a loss, the clinical significance of other findings, such as unclassified CNVs, remains to be determined. As a general rule, the significance will correlate with the amount of genomic material included, and the number and significance of the genes within that locus. The three non-mosaic trisomies were all from cases where the maternal age was 40 years or greater.

### DNA quality data

The DNA integrity, including both quality and quantity, was reflected by the array QC metrics, Distribution of Log2 Ratio Spread (DLRS) (Table 2). Three cases (18.75%) with DLRS <0.3 were listed as “good”; 10 cases (62.5%) with DLRS between 0.3 and 0.5 were listed as “poor”; and 3 (18.75%) with DLRS >0.5 as “very poor”. The mean DLRS was 0.42 ± 0.16.

### Predictors of aCGH success

Comparing those samples found to be sufficient for high-resolution aCGH, sufficient for low-resolution aCGH or insufficient, there was no difference in the median gestational age at time of pregnancy loss (8 [7-16] v 9 [8-10] v 8 [7,8], respectively), p = 0.32 (Table 3). Similarly, there was no significant difference between the median interval between specimen collection and time to processing (114 days [63-264] v 313 days [229-545] v 239 days [162-1770], respectively), p = 0.14. Nor were there any significant differences in the method of obtaining the tissue (p = 0.59), the median interval between demise and collection (p = 0.33) or the percentage of abnormal findings (p > 0.99). Insufficient results were reported faster than tests generating results at low- or high-resolution (p = 0.03).

**Table 3:** Specimen characteristics by test outcome

	**Insufficient**	**Low-resolution**	**High-resolution**	**p**
*Gestational Age*	8 [7,8]	9 [8-10]	8 [7-16]	0.32
***Collection Procedure***				0.59
*D&C*	4 (100%)	3 (60%)	9 (81.8%)	
*MVA*	0 (0%)	2 (40%)	1 (9.1%)	
*SP*	0 (0%)	0 (0%)	1 (9.1%)	
*Demise to Collection (days)*	1, n = 1	0 [0-2], n = 3	2 [2-7], n = 5	0.33
*Collection to Extraction (days)*	239 [162-1770]	313 [220-545]	114 [63-264]	0.14
*Extraction to Reporting (days)*	2 [1.5-16]	13 [9-15]	35 [13-41]	0.03
*Abnormal Finding*	-	2 (40%)	6 (54.6%)	>0.99

*Abbreviations:D&C* dilation and curettage, *MVA* manual vacuum aspiration, *SP* spontaneous passage.

## Discussion

Rescue karyotyping using aCGH on DNA extracted from paraffin embedded tissue provided clinically relevant fetal cytogenetic information in 80% of patients who had undergone either failed or no attempts at conventional karyotyping in prior losses. Though most of these abnormalities could be detected with conventional karyotyping, when that testing is unavailable for any reason, rescue karyotyping provides a critical alternative method to uncover the genomic information. While other technologies, such as fluorescence *in situ* hybridization (FISH), quantitative fluorescence polymerase chain reaction (QF-PCR), and sub-telomeric multiplex ligation-dependent probe amplification have been utilized for genetic evaluation, these methods only assay targets designated *a priori*, and not the entire genome. Microdissection of chorionic villi from a single slide followed by whole genome amplification (WGA) is technically challenging and not amenable to routine application in a clinical laboratory setting [28].

Our ability to attain this information relies on the critical fact that unlike conventional karyotyping, aCGH testing can be performed from paraffin embedded tissue blocks that are routinely prepared and archived following a D&C as part of standard hospital protocol. The process requires minimal effort from the provider, aside from procuring these slides and getting them sent for testing at a qualified center. As such, once protocols have been established for coordinating the movement of specimen slides from the original facility to the testing facility and for communicating the results to the ordering physician, the process is quite simple. This simplicity lends itself to the possibility of increased utilization in patients with RPL.

aCGH is also able to detect smaller copy number variants compared to traditional karyotyping, and other studies have already established a higher pickup rate with aCGH. In a recent study by Reddy et al. aCGH yielded results more often than traditional karyotyping (87.4% v 70.5%, p < 0.001) and provided better detection of aneuploidy or pathogenic copy-number variants (8.3 v 5.8%, p = 0.007) [25]. Despite the small number in this case series, our success rate at obtaining results is on par with these results, and we have been able to utilize this technology to obtain genomic information that would otherwise not have been available. More widespread use of this technology may expand our knowledge about CNV associated with unexplained RPL. This could provide insights to both patients and providers, and may also hone our precision in using pre-implantation genetic diagnosis and screening methods to assist these patients in completing euploid pregnancies. This information can be helpful in determining whether a more extensive and costly RPL evaluation is necessary and may also provide a definitive explanation for a pregnancy loss for those patients who require it. However, CNVs of undetermined clinical significance can also be discovered and these may not play a causal role in the miscarriage.

Though current institutional rates for traditional karyotyping is $820, and microarray testing $1600-1750 depending on resolution, this cost disparity only underscores the utility of it as described here, as a rescue method when traditional testing fails or is not performed. Further, we anticipate the cost for microarray testing to continue to fall as technology advances, while the cost of traditional G-band karyotyping will likely remain constant or even increase due to labor costs. Additionally, the patient’s insurance typically covers microarray testing in this context; this was the case for all of the patients in our cohort. In summary, aCGH testing holds potential for cost-effective use in rescue karyotyping by enabling cytogenetic evaluation of archived fetal tissue. This can provide physicians and patients with clinically relevant cytogenetic information on prior pregnancy losses. The results of this testing can help guide a physician’s decision to pursue a RPL evaluation and can provide sense of closure to patients.

## Abbreviations

aCGH: Array-based comparative genomic hybridization; CNV: Copy number variant; DLRS: Distribution of log2 ratio spread; D&C: Dilation & curettage; FFPE: Formalin-fixed paraffin-embedded; FISH: Fluorescence in-situ hybridization; IQR: Inter-quartile range; MVA: Manual vacuum aspiration; POCs: Products of conception; QF-PCR: Quantitative fluorescence polymerase chain reaction; RPL: Recurrent pregnancy loss; WGA: Whole genome amplification.

## Competing interests

The authors declare that they have no competing interests.

## Authors’ contributions

RK performed the data and statistical analysis and drafted the manuscript. ML, JS and AK carried out the molecular studies. ZW conceived and designed the experiments and helped to draft the manuscript. All authors read and approved the final manuscript.

## References

[B1] Stirrat GM Recurrent miscarriage Lancet 1990 336 673 675 10.1016/0140-6736(90)92159-F 1975862

[B2] Practice Committee of the American Society for Reproductive Medicine Definitions of infertility and recurrent pregnancy loss: a committee opinion Fertil Steril 2013 99 63 23095139 10.1016/j.fertnstert.2012.09.023

[B3] Lin CC De Braekeleer M Jamro H Cytogenetic studies in spontaneous abortion: the Calgary experience Can J Genet Cytol 1985 27 565 570 4063875 10.1139/g85-083

[B4] Nagaishi M Yamamoto T Iinuma K Shimomura K Berend SA Knops J Chromosome abnormalities identified in 347 spontaneous abortions collected in Japan J Obstet Gynaecol Res 2004 30 237 241 10.1111/j.1447-0756.2004.00191.x 15210050

[B5] Ogasawara M Aoki K Okada S Suzumori K Embryonic karyotype of abortuses in relation to the number of previous miscarriages Fertil Steril 2000 73 300 304 10.1016/S0015-0282(99)00495-1 10685533

[B6] Simpson JL Causes of fetal wastage Clin Obstet Gynecol 2007 50 10 30 10.1097/GRF.0b013e31802f11f6 17304022

[B7] Stern JJ Dorfmann AD Gutierrez-Najar AJ Cerrillo M Coulam CB Frequency of abnormal karyotypes among abortuses from women with and without a history of recurrent spontaneous abortion Fertil Steril 1996 65 250 253 8566242 10.1016/s0015-0282(16)58079-0

[B8] Sullivan AE Silver RM LaCoursiere DY Porter TF Branch DW Recurrent fetal aneuploidy and recurrent miscarriage Obstet Gynecol 2004 104 784 788 10.1097/01.AOG.0000137832.86727.e2 15458902

[B9] Fritz B Hallermann C Olert J Fuchs B Bruns M Aslan M Schmidt S Coerdt W Muntefering H Rehder H Cytogenetic analyses of culture failures by comparative genomic hybridisation (CGH)-Re-evaluation of chromosome aberration rates in early spontaneous abortions Eur J Hum Genet 2001 9 539 547 10.1038/sj.ejhg.5200669 11464246

[B10] Philipp T Philipp K Reiner A Beer F Kalousek DK Embryoscopic and cytogenetic analysis of 233 missed abortions: factors involved in the pathogenesis of developmental defects of early failed pregnancies Hum Reprod 2003 18 1724 1732 10.1093/humrep/deg309 12871891

[B11] Stephenson MD Awartani KA Robinson WP Cytogenetic analysis of miscarriages from couples with recurrent miscarriage: a case–control study Hum Reprod 2002 17 446 451 10.1093/humrep/17.2.446 11821293

[B12] Practice Committee of the American Society for Reproductive Medicine Evaluation and treatment of recurrent pregnancy loss: a committee opinion Fertil Steril 2012 98 1103 1111 22835448 10.1016/j.fertnstert.2012.06.048

[B13] Bernardi LA Plunkett BA Stephenson MD Is chromosome testing of the second miscarriage cost saving? A decision analysis of selective versus universal recurrent pregnancy loss evaluation Fertil Steril 2012 98 156 161 10.1016/j.fertnstert.2012.03.038 22516510

[B14] Foyouzi N Cedars MI Huddleston HG Cost-effectiveness of cytogenetic evaluation of products of conception in the patient with a second pregnancy loss Fertil Steril 2012 98 151 155 10.1016/j.fertnstert.2012.04.007 22748232

[B15] Gao J Liu C Yao F Hao N Zhou J Zhou Q Zhang L Liu X Bian X Liu J Array-based comparative genomic hybridization is more informative than conventional karyotyping and fluorescence in situ hybridization in the analysis of first-trimester spontaneous abortion Mol Cytogenet 2012 5 33 10.1186/1755-8166-5-33 22794168 PMC3488553

[B16] Raca G Artzer A Thorson L Huber S Modaff P Laffin J Pauli RM Array-based comparative genomic hybridization (aCGH) in the genetic evaluation of stillbirth Am J Med Genet A 2009 149A 2437 2443 10.1002/ajmg.a.33083 19876905

[B17] Harris RA Ferrari F Ben-Shachar S Wang X Saade G Van Den Veyver I Facchinetti F Aagaard-Tillery K Genome-wide array-based copy number profiling in human placentas from unexplained stillbirths Prenat Diagn 2011 31 932 944 10.1002/pd.2817 21732394 PMC3183137

[B18] Benkhalifa M Kasakyan S Clement P Baldi M Tachdjian G Demirol A Gurgan T Fiorentino F Mohammed M Qumsiyeh MB Array comparative genomic hybridization profiling of first-trimester spontaneous abortions that fail to grow in vitro Prenat Diagn 2005 25 894 900 10.1002/pd.1230 16088865

[B19] Menten B Swerts K Delle Chiaie B Janssens S Buysse K Philippe J Speleman F Array comparative genomic hybridization and flow cytometry analysis of spontaneous abortions and mors in utero samples BMC Med Genet 2009 10 89 19751515 10.1186/1471-2350-10-89 PMC2753309

[B20] Robberecht C Schuddinck V Fryns JP Vermeesch JR Diagnosis of miscarriages by molecular karyotyping: benefits and pitfalls Genet Med 2009 11 646 654 10.1097/GIM.0b013e3181abc92a 19617844

[B21] Schaeffer AJ Chung J Heretis K Wong A Ledbetter DH Lese Martin C Comparative genomic hybridization-array analysis enhances the detection of aneuploidies and submicroscopic imbalances in spontaneous miscarriages Am J Hum Genet 2004 74 1168 1174 10.1086/421250 15127362 PMC1182080

[B22] Shimokawa O Harada N Miyake N Satoh K Mizuguchi T Niikawa N Matsumoto N Array comparative genomic hybridization analysis in first-trimester spontaneous abortions with “normal” karyotypes Am J Med Genet A 2006 140 1931 1935 16906550 10.1002/ajmg.a.31421

[B23] Zhang YX Zhang YP Gu Y Guan FJ Li SL Xie JS Shen Y Wu BL Ju W Jenkins EC Brown WT Zhong N Genetic analysis of first-trimester miscarriages with a combination of cytogenetic karyotyping, microsatellite genotyping and arrayCGH Clin Genet 2009 75 133 140 10.1111/j.1399-0004.2008.01131.x 19215247

[B24] Wapner RJ Martin CL Levy B Ballif BC Eng CM Zachary JM Savage M Platt LD Saltzman D Grobman WA Klugman S Scholl T Simpson JL McCall K Aggarwal VS Bunke B Nahum O Patel A Lamb AN Thom EA Beaudet AL Ledbetter DH Shaffer LG Jackson L Chromosomal microarray versus karyotyping for prenatal diagnosis N Engl J Med 2012 367 2175 2184 10.1056/NEJMoa1203382 23215555 PMC3549418

[B25] Reddy UM Page GP Saade GR Silver RM Thorsten VR Parker CB Pinar H Willinger M Stoll BJ Heim-Hall J Varner MW Goldenberg RL Bukowski R Wapner RJ Drews-Botsch CD O'Brien BM Dudley DJ Levy B NICHD Stillbirth Collaborative Research Network Karyotype versus microarray testing for genetic abnormalities after stillbirth N Engl J Med 2012 367 2185 2193 10.1056/NEJMoa1201569 23215556 PMC4295117

[B26] Hillman SC Pretlove S Coomarasamy A McMullan DJ Davison EV Maher ER Kilby MD Additional information from array comparative genomic hybridization technology over conventional karyotyping in prenatal diagnosis: a systematic review and meta-analysis Ultrasound Obstet Gynecol 2011 37 6 14 10.1002/uog.7754 20658510

[B27] Miller DT Adam MP Aradhya S Biesecker LG Brothman AR Carter NP Church DM Crolla JA Eichler EE Epstein CJ Faucett WA Feuk L Friedman JM Hamosh A Jackson L Kaminsky EB Kok K Krantz ID Kuhn RM Lee C Ostell JM Rosenberg C Scherer SW Spinner NB Stavropoulos DJ Tepperberg JH Thorland EC Vermeesch JR Waggoner DJ Watson MS Consensus statement: chromosomal microarray is a first-tier clinical diagnostic test for individuals with developmental disabilities or congenital anomalies Am J Hum Genet 2010 86 749 764 10.1016/j.ajhg.2010.04.006 20466091 PMC2869000

[B28] Bell KA Van Deerlin PG Feinberg RF du Manoir S Haddad BR Diagnosis of aneuploidy in archival, paraffin-embedded pregnancy-loss tissues by comparative genomic hybridization Fertil Steril 2001 75 374 379 10.1016/S0015-0282(00)01703-9 11172842

[B29] Tapper J Butzow R Wahlstrom T Seppala M Knuutila S Evidence for divergence of DNA copy number changes in serous, mucinous and endometrioid ovarian carcinomas Br J Cancer 1997 75 1782 1787 10.1038/bjc.1997.304 9192982 PMC2223609

[B30] Li MM Nimmakayalu MA Mercer D Andersson HC Emanuel BS Characterization of a cryptic 3.3 Mb deletion in a patient with a ‘balanced t(15;22) translocation’ using high density oligo array CGH and gene expression arrays Am J Med Genet A 2008 146 368 375 18203177 10.1002/ajmg.a.32116 PMC2810975

